# Bloodstream Infections in Community Hospitals in the 21^st^ Century: A Multicenter Cohort Study

**DOI:** 10.1371/journal.pone.0091713

**Published:** 2014-03-18

**Authors:** Deverick J. Anderson, Rebekah W. Moehring, Richard Sloane, Kenneth E. Schmader, David J. Weber, Vance G. Fowler, Emily Smathers, Daniel J. Sexton

**Affiliations:** 1 Duke University Division of Infectious Diseases, Duke University Medical Center, Durham, North Carolina, United States of America; 2 Duke Infection Control Outreach Network, Durham, North Carolina, United States of America; 3 Center for the Study of Aging and Human Development, Duke University Medical Center, Durham, North Carolina, United States of America; 4 Department of Medicine-Geriatrics, Duke University Medical Center and Geriatric Research Education and Clinical Center (GRECC), Durham VA Medical Center, Durham, North Carolina, United States of America; 5 Department of Hospital Epidemiology, University of North Carolina Health System, Chapel Hill, North Carolina, United States of America; Columbia University, United States of America

## Abstract

**Background:**

While the majority of healthcare in the US is provided in community hospitals, the epidemiology and treatment of bloodstream infections in this setting is unknown.

**Methods and Findings:**

We undertook this multicenter, retrospective cohort study to 1) describe the epidemiology of bloodstream infections (BSI) in a network of community hospitals and 2) determine risk factors for inappropriate therapy for bloodstream infections in community hospitals. 1,470 patients were identified as having a BSI in 9 community hospitals in the southeastern US from 2003 through 2006. The majority of BSIs were community-onset, healthcare associated (n = 823, 56%); 432 (29%) patients had community-acquired BSI, and 215 (15%) had hospital-onset, healthcare-associated BSI. BSIs due to multidrug-resistant pathogens occurred in 340 patients (23%). Overall, the three most common pathogens were *S. aureus* (n = 428, 28%), *E. coli* (n = 359, 24%), coagulase-negative Staphylococci (n = 148, 10%), though type of infecting organism varied by location of acquisition (e.g., community-acquired). Inappropriate empiric antimicrobial therapy was given to 542 (38%) patients. Proportions of inappropriate therapy varied by hospital (median = 33%, range 21–71%). Multivariate logistic regression identified the following factors independently associated with failure to receive appropriate empiric antimicrobial therapy: hospital where the patient received care (p<0.001), assistance with ≥3 ADLs (p = 0.005), Charlson score (p = 0.05), community-onset, healthcare-associated infection (p = 0.01), and hospital-onset, healthcare-associated infection (p = 0.02). Important interaction was observed between Charlson score and location of acquisition.

**Conclusions:**

Our large, multicenter study provides the most complete picture of BSIs in community hospitals in the US to date. The epidemiology of BSIs in community hospitals has changed: community-onset, healthcare-associated BSI is most common, *S. aureus* is the most common cause, and 1 of 3 patients with a BSI receives inappropriate empiric antimicrobial therapy. Our data suggest that appropriateness of empiric antimicrobial therapy is an important and needed performance metric for physicians and hospital stewardship programs in community hospitals.

## Introduction

Bloodstream infections (BSIs) are a leading cause of suffering and death in the US. As many as 250,000 BSIs occur each year [1], with a mortality rate of 35% and costs of up to $37,000 per case [2], [3]. In fact, BSI was one of the top causes of death in the US in 2008, leading to more than 35,000 deaths [4].

The majority of healthcare in the US is performed in smaller, non-teaching community hospitals._ENREF_4 The mean size of hospitals in the US was 160 beds in 2009, and 72% of hospitals had fewer than 200 beds [4]._ENREF_4 Of the 39 million hospital discharges in the US in 2010, 19.9 million (51%) were from non-teaching facilities [5]. Our understanding about the causes and risk factors for BSI in these community hospitals, however, is alarmingly inadequate. Prior studies on BSIs in community hospitals have been limited to specific organisms [6], [7], single institutions, intensive care units [8] and/or patients admitted prior to the emergence and spread of epidemiologically important multidrug-resistant organisms [9]–[14]. Even less is known about the impact of antibacterial resistance in community hospitals [15].

Limited available data suggest that patients with BSI are less likely to receive appropriate therapy in community hospitals as compared to tertiary care hospitals [15]. These treatment trends are important, as inappropriate empiric therapy leads to a 60% increase in mortality [16].

We undertook this study in order to 1) describe the epidemiology of BSIs in a network of community hospitals and 2) determine risk factors for inappropriate therapy for BSIs in community hospitals.

## Materials and Methods

### Ethics Statement

This study was reviewed and approved by the Institutional Review Board (IRB) of Duke University Health System. Participating community hospitals deferred to the Duke IRB (n = 5), or reviewed and approved the study via their local IRB (n = 4). Written patient consent was waived by all sites.

### Study Design and Participating Hospitals

This retrospective cohort study included adult subjects admitted to nine community, non-academic hospitals in North Carolina and Virginia from January 1, 2003, through December 31, 2006 (hereafter, the “study period”). The median bed size of participating hospitals was 151 (range 102–355) beds. All hospitals were members of the Duke Infection Control Outreach Network (DICON) [17]. In brief, DICON provides infection control consulting, data feedback, education, and quality improvement services to 42 hospitals in the southeastern US.

### Patient Selection

We reviewed data from all consecutive positive blood cultures from participating hospitals' microbiology laboratory databases during the study period. Patients were randomly selected from the overall cohort. Our goal enrollment was 1,400 patients. Assuming that 35% of patients would receive inappropriate therapy [15], [18], the study had >80% power to identify a risk factor for inappropriate therapy that was 8% prevalent among patients who received inappropriate therapy compared to 4% prevalent among patients who received appropriate therapy (alpha = 0.05). Random sampling by random number generator was performed to ensure that subjects were included equally from all four years of the study period.

Trained data abstractors collected all patient data by chart review, including detailed clinical, demographic, microbiologic, treatment data, and outcomes. A standardized data collection tool, data dictionary, and standard operating procedure were created prior to data abstraction. Any patient with a bloodstream infection was considered for inclusion. Selected patients were included in the database once. That is, if a patient's second cases of BSI was randomly selected for inclusion, it was excluded and a new patient was randomly selected.

### Definitions

BSIs were defined using modified Centers for Disease Control and Prevention (CDC) criteria: ≥ 1 positive blood culture for all bacterial pathogens except common skin contaminants including Enterococci which require ≥ 2 positive blood cultures within 48 hours. Onset of infection was defined as the time of the first positive blood culture. “Appropriate empiric antimicrobial therapy” was defined as receipt of an antimicrobial agent with *in vitro* activity against the infecting organism within 24 hours after the onset of infection. Location of acquisition was defined using CDC criteria [19] as follows: 1) “community-onset, healthcare-associated” was defined as a BSI occurring <48 hours after admission plus the presence of ≥1 of the following healthcare risk factors: prior hospitalization, surgery, dialysis, or residence in a long-term care facility in the 12 months preceding the BSI, or the presence of an invasive device; 2) “community-acquired” was defined as a BSI occurring <48 hours after admission without one of the above healthcare risk factors; and 3) “hospital-onset, healthcare-associated” was defined as a BSI that occurred ≥ 48 hours after hospital admission.

Multidrug resistance was defined using consensus definitions [20]. Charlson comorbidity index and McCabe score were used to measure baseline severity of illness at the time of admission [21], [22]._ENREF_6 APACHE II score was calculated to measure severity of illness at the time of infection [23]. Functional status was measured as independent or not independent for 5 activities of daily living (ADLs) using the Katz criteria [24]._ENREF_22 A binary variable for functional status was created to measure severe disability which was defined as lack of independence with ≥3 ADLs. Finally, a secondary BSI was defined as a BSI that occurred as a result of a microbiologically-diagnosed infection from another body site, excluding central venous catheters.

### Statistics

Rates were calculated as number of BSIs/1,000 patient-days. Standard descriptive statistics were used for categorical and continuous variables. Bivariable analyses were performed using the Student's t-test or Wilcoxon rank sums test, as appropriate, for continuous variables and the **χ** ^2^ test for categorical variables. All tests were two-tailed; a p-value ≤0.05 was considered to be significant for all tests. Statistical analyses were performed using SAS v9.3 (Cary, NC).

The main outcome of interest was “failure to receive appropriate empiric antimicrobial therapy.” For simplicity, patients who experienced the outcome will hereafter been labeled as “cases.” Our *a priori* covariate of interest was location of acquisition. Logistic regression models were created to identify independent variables associate with the main outcome. For each model, candidate variables were included in the full model if p<0.2 in bivariable analysis. No correction for multiple comparisons was made to ensure that our models were inclusive of all appropriate candidate variables. Variables considered for inclusion in the models were assessed for missing data. All evaluated variables were missing fewer than 5% of the time. Thus, missing data for these variables were imputed using unconditional imputation: imputation of the mean for continuous variables or the mode for categorical variables [25]. Effect measure modification was evaluated using interaction terms with the co-variate of interest. The Likelihood ratio test was used to determine the most parsimonious model if group variables (e.g., Charlson score) and specific components of the group variable (e.g., diabetes) were candidate variables. Candidate variables were assessed for co-linearity, and co-linear variables were removed, as necessary. The final model was created using manual backwards selection. During this process, removed variables were assessed for confounding. A variable was considered to be a potential confounder if the β-estimate for any variable changed >10% after its removal. Finally, a class variable for “hospital” was included in the model, and the generalized estimating equation method was used to account for clustering of the outcome of interest within individual hospitals.

The first and primary model was created using only variables available to treating clinicians at the onset of infection. The second model was created in a similar fashion but candidate variables also included variables available after the onset of infection (e.g., multidrug resistance and polymicrobial infection).

## Results

### Epidemiology of BSIs in Community Hospitals

A total of 5,124 patients had a BSI during 1,371,467 patient days during the study period. The median rate of BSI per hospitals was 3.5 BSIs/1000 patient days (IQR 3.0–4.2). The study cohort consisted of a sample of 1,470 (29%) unique patients who were randomly selected for inclusion in the study. Patients randomly selected for inclusion had similar organism distribution to patients not selected.

Patients were generally older (mean age 65.3 ± 17.2) and Caucasian race (n = 759, 52%) but a high proportion of patients were African-American race (n = 680, 46%) (Table 1). A total of 1,091 (74%) patients were admitted from home, and 312 (21%) were admitted from a nursing home; 745 (51%) patients had been hospitalized in the prior 12 months.

**Table 1:** Patient Demographics, Co-morbidities, and Hospitalization Information among 1,470 patients with bloodstream infection (BSI) in nine community hospitals, 2003–2006^a^^,^ b.

	Total cohort	Patients who did not receive appropriate empiric antimicrobial therapy	Patients who received appropriate empiric antimicrobial therapy	P-value
	N = 1470	N = 542	N = 906	
	n (%)	n (%)	n (%)	
***Patient Demographics***				
Age – mean (STD)	65.3 (17.2)	65.9 (17.6)	64.9 (17.0)	0.36
Female Sex	765 (52)	285 (53)	463 (51)	0.59
Race				0.64
White	759 (52)	276 (51)	473 (52)	
Black	680 (46)	251 (47)	417 (46)	
Other	19 (1)	9 (1)	10 (1)	
Married	611 (44)	222 (41)	380 (42)	0.71
Insurance				
Medicare	1025 (70)	369 (69)	639 (71)	0.38
Medicaid	111 (8)	48 (9)	62 (7)	
Private	209 (15)	78 (15)	127 (14)	
None	99 (7)	32 (6)	67 (7)	
BMI – mean (STD)	27.9 (8.9)	27.7 (8.5)	28.0 (9.2)	0.5
***Co-morbid conditions at Admission***				
Need assistance with any ADL	844 (57)	334 (62)	498 (55)	0.01
Need assistance with ≥ 3 ADL	384 (26)	183 (34)	195 (22)	<0.0001
McCabe score at admission				0.36
1	279 (19)	114 (21)	162 (18)	
2	805 (55)	295 (55)	494 (55)	
3	369 (25)	126 (23)	240 (27)	
On immunosuppressive medication				0.55
Corticosteroid	113 (8)	43 (8)	67 (8)	
Non-corticosteroid	27 (2)	12 (2)	15 (2)	
Both	7 (1)	4 (1)	3 (1)	
***Comorbidities***				
Charlson score (median, IQR)	2 (1–4)	2 (1–4)	2 (1–3)	0.02
Charlson score >3 (binary)	405 (28)	178 (33)	223 (25)	0.0007
Diabetes	626 (43)	243 (45)	373 (41)	0.17
Diabetes with end organ damage	9 (1)	6 (1)	3 (1)	
Myocardial infarction	329 (22)	125 (23)	200 (22)	0.66
Congestive heart failure	313 (21)	125 (23)	184 (20)	0.22
Peripheral vascular disease	218 (15)	96 (18)	121 (13)	0.03
Cerebrovascular disease	299 (20)	106 (20)	184 (20)	0.73
Dementia	219 (15)	99 (18)	118 (13)	0.007
Chronic obstructive pulmonary disease	273 (19)	108 (20)	159 (18)	0.26
Connective tissue disease	17 (1)	8 (1)	9 (1)	0.41
Peptic ulcer disease	206 (14)	90 (17)	113 (12)	0.03
Hemiplegia	33 (2)	17 (3)	14 (2)	0.04
Liver disease	108 (7)	41 (8)	65 (7)	0.78
Chronic Kidney Insufficiency	435 (30)	161 (30)	268 (30)	0.96
End stage renal disease requiring dialysis	194 (13)	67 (12)	123 (14)	0.51
Hemodialysis	187 (12)	64 (12)	119 (13)	0.46
Peritoneal dialysis	7 (1)	3 (1)	4 (1)	
History of malignancy	309 (21)	116(21)	189 (21)	0.81
Metastatic disease	46 (3)	19 (4)	27 (3)	0.58
HIV/AIDS	31 (2)	10 (2)	21 (2)	0.55
Transplant	11 (1)	4 (1)	7 (1)	1.0
Tobacco use ongoing	358 (24)	129 (24)	228 (25)	0.59
Alcohol use ongoing	208 (14)	67 (12)	138 (15)	0.14
***Infection risks***				
AICD or Pacemaker present at admission	69 (5)	25 (5)	44 (5)	0.84
Documented decubitus at admission	232 (16)	91 (17)	135 (15)	0.32
Intravascular catheter present at admission	288 (20)	114 (21)	167 (19)	0.24
Urinary catheter present at admission	160 (11)	67 (12)	90 (10)	0.15
PEG present at admission	87 (6)	35 (7)	51 (6)	0.62
History of resistant organism	96 (7)	37 (7)	57 (6)	0.62
Duration of hospitalization prior to BSI (days) median (IQR)	0 (0–0.9)	0 (0–1)	0 (0–0.7)	0.44
***Hospitalization characteristics***				
Admitting service				0.90
Medical	1302 (89)	481 (89)	802 (89)	
Surgical	101 (7)	36 (7)	63 (7)	
Other	64 (4)	22 (4)	41 (5)	
Admission source				<0.001
Home	1091 (74)	364 (67)	712 (79)	
Nursing Home	312 (21)	140 (26)	166 (18)	
Rehabilitation facility	13 (1)	8 (1)	5 (1)	
Other Hospital	33 (2)	20 (4)	12 (1)	
Other	19 (2)	8 (1)	11 (1)	
Admitted from facility	358 (24)	168 (31)	183 (20)	<0.0001
Hospitalized in prior 12 months	745 (51)	304 (56)	430 (48)	0.002

a Abbreviations: STD = standard deviation; ADL = activity of daily living; BMI = body mass index; IQR = interquartile range; AICD = automated internal cardiac defibrillator; PICC = peripherally inserted central catheter; PEG = percutaneous gastrostomy; MRSA = methicillin-resistant Staphylococcus aureus; ESBL = extended spectrum beta-lactamase producing organism; VRE = vancomycin-resistant enterococci.

b Missing data: Age (n = 1), sex (n = 5), race (n = 12), marital status (n = 69), insurance (n = 22), BMI (n = 88), ambulation (n = 1), bathing (n = 1), dressing (n = 1), bowel incontinence (n = 4), urine incontinence (n = 3), feeding (n = 1), McCabe score (n = 17), immunosuppressive medications (n = 28), tobacco use (n = 5), alcohol use (n = 8), pacemaker present (n = 12), documented decubitus (n = 13), intravascular catheter (n = 10), urinary catheter (n = 12), percutaneous gastrostomy tube (n = 13), history of infection or colonization due to a resistant organism (n = 40), admitting service (n = 3), admission source (n = 2), history of hospitalization in prior 12 months (n = 5).

**Figure 1:**
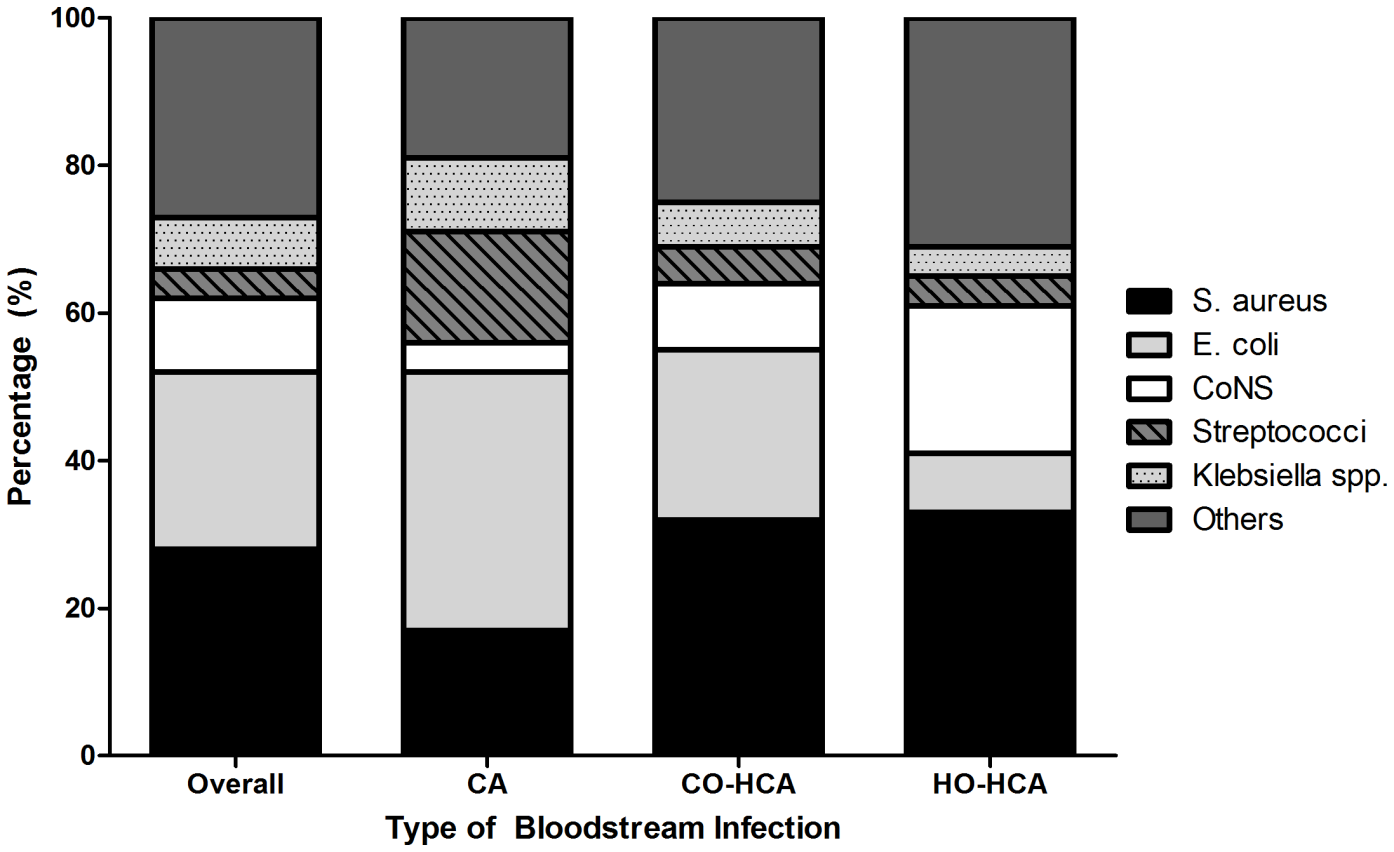
Distribution of pathogens based on location of acquisition of bloodstream infection (BSI) among 1,470 patients admitted to 9 community hospitals, 2003–2006. [FOOTNOTE] * CA = community-acquired, CO-HCA = community-onset, healthcare-associate, HO-HCA; hospital-onset, healthcare-associated, CoNS = coagulase negative Staphylococci.

The majority of BSIs were community-onset, healthcare associated (n = 823, 56%; Table 2); 432 (29%) patients had community-acquired BSI, and 215 (15%) had hospital-onset, healthcare-associated BSI. BSIs due to multidrug-resistant pathogens occurred in 340 patients (23%). A total of 1,514 separate pathogens were identified during the 1,470 BSIs (Table 3). Overall, the most common pathogen was *S. aureus* (n = 428, 28%), though type of infecting organism varied by location of acquisition (Figure 1). The most common multidrug-resistant pathogens were methicillin resistant *Staphylococcus aureus* (MRSA) (n = 203, 13%), E. coli (n = 79, 5%), coagulase-negative staphylococci (n = 51, 3%), and *Pseudomonas aeruginosa* (n = 8, 0.5%).

**Table 2:** Infection and Treatment Data for 1,470 patients with bloodstream infection (BSI) in nine community hospitals, 2003–2006^a^.

	Total cohort	Patients who did not receive appropriate empiric antimicrobial therapy	Patients who received appropriate empiric antimicrobial therapy	P-value
	N = 1470	N = 542	N = 906	
	n (%)	n (%)	n (%)	
Documented infection in past year	273 (20)	106 (20)	167 (19)	0.58
***BSI DATA***				
Type of BSI				0.57
Secondary	303 (21)	107 (20)	194 (21)	
Urine	173 (12)	61 (11)	111 (12)	
Wound	33 (2)	13 (2)	20 (2)	
Pneumonia	59 (4)	21 (4)	36 (4)	
Other	38 (3)	13 (2)	25 (3)	
No secondary source identified or due to central venous catheter	1163 (79)	434 (80)	710 (79)	
Location of acquisition				<0.001
Community-associated	432 (29)	126 (23)	302 (33)	
Community-onset, healthcare-associated	823 (56)	314 (58)	501 (55)	
Hospital-onset, healthcare-associated	215 (15)	102 (19)	103 (11)	
In intensive care unit prior to BSI	87 (6)	40 (7)	44 (5)	0.05
Central line present at BSI	289 (20)	116 (22)	163 (19)	0.08
*Organism*				
Multidrug Resistant	340 (23)	170 (32)	170 (19)	<0.0001
Resistant Gram negative pathogen	99 (7)	37 (7)	62 (7)	0.90
Resistant Gram positive pathogen	241 (17)	133 (25)	108 (12)	<0.0001
Polymicrobial	60 (4)	18 (3)	42 (5)	0.22
APACHE score at time of BSI – mean (STD)	14.5 (4.9)	14.5 (5.0)	14.4 (4.8)	0.74

a Missing data: type of BSI (n = 3), location of acquisition of BSI (n = 4), central line present at BSI (n = 51).

**Table 3:** Microbiology of 1,514 isolates from 1,470 patients with bloodstream infection in community hospitals^a^.

	Total Pathogens
	N = 1,514
	n (%)
Gram positive organisms	823 (54)
* Staphylococcus aureus*	428 (28)
Methicillin susceptible	225 (15)
Methicillin resistant	203 (13)
Coagulase negative staphylococci	148 (10)
* Enterococcus*	52 (3)
Group B *Streptococcus*	43 (3)
Group A *Streptococcus*	19 (1)
Viridans group streptococci	10 (1)
Gram negative organisms	660 (44)
* E. coli*	359 (24)
* Klebsiella*	100 (7)
* Pseudomonas*	51 (3)
* Proteus*	58 (4)
* Enterobacter*	30 (2)
* Serratia*	13 (1)
* Citrobacter*	11 (1)
* Acinetobacter*	10 (1)
Anaerobes	14 (1)
*Candida*	10 (1)

a Isolates that led to <10 bloodstream infections are not included.

The in-hospital mortality rate was 18% (n = 264; Table 4). Patients with any prior healthcare exposure were almost 3-fold more likely to die than patients without prior healthcare exposure (OR = 2.78 95% CI 1.94–4.00). The in-hospital mortality rate was 21% (n = 170) for patients with community-onset, healthcare-associated infection and 26% (n = 55) for hospital-onset, healthcare-associated infection.

**Table 4:** Outcomes data for 1,470 patients with bloodstream infection (BSI) in nine community hospitals, 2003–2006^a^.

	Total cohort	Patients who did not receive appropriate empiric antimicrobial therapy	Patients who received appropriate empiric antimicrobial therapy	P-value
	N = 1470	N = 542	N = 906	
	n (%)	n (%)	n (%)	
In week following BSI,				
Admitted to intensive care unit^b^	346 (26)	122 (25)	224 (27)	0.45
Central venous catheter placed	310 (21)	118 (22)	187 (21)	0.62
Intubated	183 (13)	72 (13)	106 (12)	0.39
On pressors	184 (13)	63 (12)	118 (13)	0.44
Procedure performed for BSI	170 (12)	64 (12)	102 (11)	0.75
Incision and drainage	79 (5)	31 (6)	47 (5)	
Prosthesis removal	10 (1)	4 (1)	6 (1)	
Other surgery	75 (5)	27 (5)	45 (5)	
Hospital duration - days				
Total – median (IQR)	7 (4–12)	7 (4–12)	7 (4–11)	0.04
Following BSI – median (IQR)	6 (4–11)	7 (3–12)	6 (4–10)	0.24
PICC placed for outpatient IV antibiotics	110 (8)	53 (10)	57 (6)	0.02
Died in hospital	264 (18)	106 (20)	152 (17)	0.18
Discharge status				0.01
Home	665 (46)	207 (38)	450 (50)	
Home Health	103 (7)	44 (8)	59 (7)	
Rehabilitation center	52 (4)	20 (4)	31 (3)	
Nursing Home	283 (19)	122 (23)	155 (17)	
Tertiary care hospital	77 (5)	31 (6)	45 (5)	
Left hospital against medical advice	6 (1)	3 (1)	3 (1)	
Other	15 (1)	7 (1)	8 (1)	
Readmitted within 90 days	390 (27)	154 (29)	231 (26)	0.24
Returned to Emergency Room within 90 days	347 (25)	135 (26)	209 (25)	0.53

a Missing data: Week following BSI outcomes (n = 9), procedures after BSI (n = 24), PICC placement (n = 22), discharge status (n = 5), readmitted within 90 days (n = 9), returned to ED (n = 85).

b Excludes 87 patients in ICU prior to BSI.

### Predictors of Failure to Receive Appropriate Empiric Antimicrobial Therapy

#### Bivariable analysis

Inappropriate empiric antimicrobial therapy was given to 542 (38%) patients (hereafter, “cases”). The proportion of patients who recieved inappropriate therapy varied by hospital (median = 33%, range 21–71%). In bivariable analysis, cases were more likely than non-cases to require assistance with any ADL (62% v. 55%, p = 0.01), ≥3 ADLs (34% v. 22%, p<0.001), and had a higher median Charlson score (p = 0.02) (Table 1).

Cases were more likely to have prior healthcare exposure than non-cases (p = 0.0002). More specifically, cases were more frequently admitted from a nursing facility (p<0.001) or hospitalized in the prior 12 months (p = 0.002). Location of acquisition differed between the two groups (p<0.001); cases had more hospital-onset, healthcare-associated and less community-acquired BSI than non-cases. Cases were more likely to have an infection with a multidrug-resistant Gram positive organism (p<0.001).

Unadjusted in-hospital mortality was higher among cases compared to non-cases, but the difference was not statistically significant (p = 0.18; Table 4). Cases had longer total duration of hospitalization (p = 0.04) and were more likely to require PICC placement for outpatient IV antimicrobial therapy (p = 0.02). In addition, cases had different discharge destinations than non-cases (p = 0.01). The reasons for this difference may have been related to previously noted differences in admission sources (ie, more cases were admitted from facilities). In order to investigate this further, we performed a sub-analysis limited only to patients admitted from home (n = 1,091). In this group, a smaller proportion of cases were discharged back to home compared with non-cases (52% v. 62%, p = 0.003).

#### Multivariate Analysis

Multivariate logistic regression identified several factors independently associated with failure to receive appropriate empiric antimicrobial therapy, including hospital where the patient received care (p<0.001), assistance with ≥3 ADLs (p = 0.005), and Charlson score (p = 0.05; Table 5). In addition, community-onset, healthcare-associated infection (p = 0.01) and hospital-onset, healthcare-associated infection (p = 0.02) were associated with failure to receive appropriate empiric antimicrobial therapy using community-acquired infection as reference. Important interaction was observed between Charlson score and location of acquisition.

**Table 5:** Logistic Regression Model^a^ to Identify Variables Independently Associated with Failure to Receive Appropriate Empiric Antimicrobial Therapy.

Variable	Odds Ratio (95% CI)	p-value
**MODEL 1**		
Location of acquisition		
Community-acquired	Referent	
Community-onset, healthcare-associated	*^b^	
Charlson score 0–1 (low co-morbidity)	1.69 (1.12–2.57)	0.01
Charlson score 2–4 (moderate co-morbidity)	1.49 (1.06–2.08)	0.02
Charlson score ≥5 (severe co-morbidity)	1.30 (0.79–2.15)	0.30
Hospital-onset, healthcare-associated	*^b^	
Charlson score 0–1 (low co-morbidity)	2.18 (1.17–4.06)	0.02
Charlson score 2–4 (moderate co-morbidity)	2.72 (1.66–4.46)	<0.001
Charlson score ≥5 (severe co-morbidity)	3.39 (1.34–8.54)	0.01
Require assistance with ≥3 ADLs	1.41 (1.12–1.79)	0.005
Charlson score	*^b^	0.05
Hospital	*^c^	<0.001
**MODEL 2**		
Location of acquisition		
Community-acquired	Referent	
Community-onset, healthcare-associated	*^b^	
Charlson score 0–1 (low co-morbidity)	1.60 (1.05–2.45)	0.03
Charlson score 2–4 (moderate co-morbidity)	1.37 (0.97–1.93)	0.07
Charlson score ≥5 (severe co-morbidity)	1.17 (0.74–1.85)	0.5
Hospital-onset, healthcare-associated	*^b^	
Charlson score 0–1 (low co-morbidity)	1.98 (1.06–3.71)	0.03
Charlson score 2–4 (moderate co-morbidity)	2.59 (1.53–4.38)	0.0004
Charlson score ≥5 (severe co-morbidity)	3.39 (1.17–9.82)	0.02
Require assistance with ≥3 ADLs	1.32 (1.04–1.69)	0.02
Charlson score	*^b^	0.07
Hospital	*^c^	<0.001
Infection due to a multidrug-resistant organism	2.17 (1.48–3.18)	<0.0001

a Model included a generalized effect estimate to account for clustering among hospitals and included the following confounders: admission from a facility (p = 0.17), presence of a central line at the time of BSI (p = 0.53), in the ICU prior to BSI onset (p = 0.59), and presence of a Foley catheter at the time of admission (p = 0.82).

b Two interaction terms were included in the model: Interaction between Charlson score and community-onset, healthcare associated location of acquisition (0.04) and interaction between Charlson score and hospital-onset, healthcare associated location of acquisition. No specific effect measure available for these variables due to interaction.

c Multi-level variable, therefore no effect measure available.

We then created a three part variable for Charlson score to better describe this interaction: few or no co-morbidities (Charlson score ≤1), moderate co-morbidities (2–4), and severe co-morbidity (≥5). Among patients with community-onset, healthcare-associated BSI, the most likely to fail to receive appropriate empiric antimicrobial therapy had no or few co-morbidities (OR = 1.69, 95% CI 1.12–2.57; p = 0.006) despite having lower acuity of illness than patients with moderate or severe co-morbidities (APACHE II score [IQR]: 13 [10]–[16] vs. 15 [12]–[19] vs. 15 [12]–[18], p<0.0001). All patients with hospital-onset, healthcare-associated infection were at high risk of receiving inappropriate empiric antimicrobial therapy, though patients with severe co-morbidities were at highest risk (OR = 3.39, 95% CI 1.34–8.54; p = 0.01).

A second model was created to determine if inclusion of the presence of drug-resistance, a variable not available to clinicians when they prescribed empiric antimicrobial therapy, altered any of the independent predictors identified in Model 1. Although infection due to a multidrug-resistant organism was strongly associated with failure to receive appropriate empiric antibiotic therapy (OR = 2.17, 95% CI 1.48–3.18, p<0.001), the majority of the predictors identified in Model 1 remained independently associated with failure to receive appropriate empiric antibiotic therapy even with adjustment for MDR (Table 5). The exception to this trend was Charlson score, for which the p-value changed from 0.05 to 0.07.

## Discussion

The paucity of data on BSIs in community hospitals occurs despite the fact that the majority of health care in the US is provided in this setting. In the current study, we used a large cohort of patients with BSI from 9 community hospitals to identify key aspects of the epidemiology of BSIs and risk factors for inappropriate therapy in patients in community hospitals. Key findings from our study included the following: 1) BSIs in community hospitals are typically community-onset, healthcare-associated infections; 2) the location of acquisition had a major impact on the causative organisms; 3) empiric antimicrobial treatment for patients with BSI in community hospitals is incorrect in approximately 1 of 3 patients; and 4) groups with the highest risk of receiving inappropriate empiric therapy include a) patients with hospital-onset, healthcare associated infections b) patients with community-onset, healthcare-associated infections and few co-morbidities, and c) patients with impaired functional status.

Healthcare exposure preceded the onset of BSI in almost 3 of every 4 patients in our cohort. For example, the majority of patients in our study cohort had central venous lines, had invasive devices present at the time of BSI, were elderly, and/or required assistance with activities of daily living. In fact, the majority of BSIs were community-onset, healthcare associated infections (56%). Authors of a recent multicenter study of 7 academically-affiliated hospitals concluded that community-onset, healthcare associated BSIs were 2-times more frequent than community-acquired BSIs and associated with 3-fold higher mortality [26]. Similarly, authors of a review of BSI data from 59 hospitals demonstrated that 62% of BSIs followed some type of healthcare exposure and the majority of BSISs (55%) were community-onset, healthcare-associated [27]. These findings and ours suggest that prevention efforts aimed at hospital-acquired BSI are less likely to be as effective or impactful as efforts to promptly and correctly provide effective empiric therapy for healthcare-exposed patients from the community.

The microbiology of BSIs in community hospitals has changed over the last few decades. *S. aureus* was the most common cause of BSI in our study cohort. In contrast, *E. coli* was the most common cause of BSI in community hospitals prior to 2000 [10], [12], [14]. Further analysis of our data showed that *S. aureus* was the most common pathogen in patients with community-onset, healthcare-associated BSI and hospital-onset, healthcare-associated BSI, but *E. coli* remained the most common pathogen among patients with community-acquired BSIs. Thus, the observed emergence of *S. aureus* may be related to the increasing frequency of complex outpatient medical care and the presence of indwelling devices prior to BSI.

In addition, patients in our study cohort more frequently had infections due to multi-drug resistant (MDR) pathogens than in previous studies. For example, three prior studies showed that the frequency of MRSA as a cause of BSI in community pathogens was 5-10 times lower (13% vs 1–3%) [9], [11], [12]. Overall, 23% of patients had BSIs due to MDR pathogens in our cohort. Similar changes in organisms and antimicrobial resistance have been observed in tertiary care centers previously [28]–[30], suggesting that BSIs in community hospitals are increasingly similar to BSIs seen in tertiary care centers.

Approximately 1 of every 3 patients with BSI in our study failed to receive appropriate empiric antimicrobial therapy. This proportion of inappropriate therapy is higher than in previously published studies from community hospitals. Approximately 20% of patients with BSI failed to receive appropriate empiric antimicrobial therapy prior to 2000 [11], [12]. In contrast, results from this study are similar to our recently published cohort of patients in community hospitals with MRSA bacteremia [15] and data published from tertiary care centers [18], [31]–[33]. Thus, this finding may be due to the higher prevalence of multidrug-resistant pathogens in participating community hospitals and/or suggestive of increasingly complex patients presenting to community hospitals.

Recent healthcare exposure, the need for assistance with ADLs, and infection due to a multidrug-resistant pathogen were independent predictors for failure to receive appropriate empiric therapy in this study. One counterintuitive predictor in our study cohort, however, deserves special emphasis: patients with few co-morbidities and community-onset, healthcare-associated BSI were at an increased risk of receiving inappropriate empiric antimicrobial therapy. We observed that this group had lower APACHE II scores and was less acutely ill at the time of onset of their bloodstream infection. Reasons for this finding are uncertain, although we theorize that treating clinicians were less suspicious of a severe illness such as BSI. Thus, they may have been less aggressive in administering any therapy or broad-spectrum antimicrobial therapy.

Our study has several important limitations. First, our retrospective design may have led to selection and/or misclassification bias. We addressed these issues by randomly selecting patients from the overall cohort and by training data abstractors in the use of a standard case review form with variable definitions. Second, our study involves patients admitted from 2003 to 2006. Our study, however, represents the largest, most detailed study on BSIs in community hospitals to date and provides a much needed update. In addition, we believe the issue of inappropriate empiric antimicrobial therapy has likely worsened over the last few years, as resistance rates and the number of overall BSIs have increased [34]; thus, our study may actually underestimate the problem of inappropriate empiric antimicrobial therapy. Third, our randomization approach may have led to selection bias. Our cases, however, had similar organism distribution as patients not selected by randomization. Fourth, we did not correct for multiple statistical comparisons. Thus, some of our “significant” statistical tests may be due to Type I error. The intent of this analysis, however, was to be exploratory and inclusive. Our findings will require confirmation in further studies. Finally, our multicenter study may not be generalizable to tertiary care centers.

In summary, our large, multicenter study provides the most complete picture of BSIs in community hospitals in the US to date. The types of BSIs seen in community hospitals have changed. Community-onset, healthcare-associated BSI is most common, and *S. aureus* is the most common cause. This shift has led to important ramifications. One of every 3 patients with a BSI receives inappropriate empiric antimicrobial therapy. Thus, our data confirm that appropriateness of empiric antimicrobial therapy is an important and needed performance metric for physicians and hospital stewardship programs [35]._ENREF_24 Risk of inadequate therapy is highest among patients with healthcare exposure and the disabled. Clinicians in community hospitals must identify these important risk factors when choosing antibiotic therapy, particularly given the adverse outcomes associated with inadequate therapy. We believe that most risk factors could be easily discernible using electronic data; efforts must be made to ensure that others, such as ADLs, are routinely included in electronic data. Ultimately, we believe an intervention whereby physicians are automatically notified of these risk factors when choosing empiric antimicrobial therapy is needed, particularly among community hospitals in the US.
